# The diagnostic and prognostic value of miR-188-5p in intracranial aneurysm (IA) and its potential regulatory mechanism

**DOI:** 10.1186/s41065-025-00593-3

**Published:** 2025-11-07

**Authors:** Liujia Ma, Lei Shi, Wenjie Tang

**Affiliations:** 1 Department of Neurosurgery, Yan’an University Affiliated Hospital, Yan’an, 716000 China; 2 Department of Neurosurgery, Suzhou Xiangcheng People’s Hospital, Suzhou, 215131 China; 3 Department of Neurology 2, The Fifth People’s Hospital of Jinan, No. 24297, Jingshi Road, Jinan, 250022 China

**Keywords:** Intracranial aneurysm, MiR-188-5p, IL6ST, Diagnosis, Prognosis

## Abstract

**Background and objectives:**

As the etiology of intracranial aneurysm (IA) remains uncertain and unruptured IA management continues to be debated, investigating biomarkers of the disease remains critical. This study thus evaluated the involvement of miR-188-5p in IA diagnosis, prognosis, and development to advance understanding of IA pathophysiology and treatment strategies.

**Materials and methods:**

A case-control study involving 73 IA patients and 79 healthy controls was conducted to assess the diagnostic and prognostic value of miR-188-5p in IA. A PDGF-BB-induced VSMC dedifferentiation model was constructed to explore the mechanisms. The qRT-PCR was employed to test the expression of biomolecules, while dual luciferase reporter assays were performed to ensure biomolecule interaction.

**Results:**

The serum expression of miR-188-5p was relatively higher in IA patients than in healthy controls. High serum expression of miR-188-5p exhibited both diagnostic utility for IA detection and predictive capacity for assessing rupture risk. MiR-188-5p inhibited α-SMA and SM22α expression, promoted MMP-2 and MMP-9 expression, and facilitated oxidative stress and proinflammatory cytokine expression in phenotypically switched VSMCs. MiR-188-5p negatively regulated IL6ST expression in phenotypically switched VSMCs. IL6ST mediated the modification of miR-188-5p in phenotypically switched VSMCs.

**Conclusion:**

MiR-188-5p was a biomarker for IA and its rupture. MiR-188-5p might assist IA progression by inducing VSMC phenotypic switching and cell damage. MiR-188-5p affected VSMCs by downregulating IL6ST. MiR-188-5p might be the potential target for predicting and controlling the development of IA.

## Introduction

With a prevalence of approximately 3% in the general population, intracranial aneurysm (IA) remains the most formidable cerebrovascular disorder due to its unpredictable rupture risk [1, 2]. In addition, IA is the first cause of subarachnoid hemorrhage (SAH), which can lead to a high risk of mortality in IA patients [3]. Therefore, the early identification of the disease is critical to decrease rupture risk and improve clinical outcomes [4]. Furthermore, considerable controversy persists regarding whether conservative management or interventional treatment is the appropriate therapeutic approach for unruptured IAs [5]. Identifying novel therapeutic targets capable of effectively modulating the progression of the disease also represents a critical avenue for improving clinical outcomes.

MicroRNAs (miRNAs) represent a class of small (~ 22-nucleotide) non-coding RNAs that post-transcriptionally regulate gene expression through base-pairing with the 3’ untranslated regions (3’UTRs) of target mRNAs [6, 7]. This interaction induces translational inhibition or mRNA destabilization, thereby enabling miRNAs to be involved in complex regulatory networks across diverse physiological and pathological processes [8, 9]. Among all the miRNAs, emerging evidence demonstrates that miR-188-5p plays a pivotal role in angiogenic processes, which further assist the pathogenesis of various vasoproliferative disorders [10]. In the context of IA, miR-188-5p has emerged as a promising biomarker associated with disease progression [11]. Consistent with its role in IA, miR-188-5p suppression showed protective effects against abdominal aortic aneurysm (AAA) progression [12]. Although preliminary investigations have indicated the potential diagnostic utility of miR-188-5p in IA, its exact diagnostic accuracy and prognostic significance remain to be evaluated. Moreover, there is currently no information regarding the fundamental biological role of the biomolecule in IA development. It is warranted to conduct a coordinated investigation on the association between miR-188-5p and IA.

Regarding genetic factors associated with aneurysm development, interleukin 6 cytokine family signal transducer (IL6ST) emerges as a critical player in vascular smooth muscle cell (VSMC) phenotypic transition. The IL6ST functions as a pleiotropic signal transducer for multiple cytokines, such as interleukin-6 (IL-6) and oncostatin-M (OSM) [13]. IL-6 contributes to vascular remodeling by regulating VSMC proliferative responses, which are critical for the progression of atherosclerosis, a disease that is one of the reasons for aneurysm formation [14]. The OSM-mediated vascular endothelial growth factor (VEGF) up-regulation in VSMC contributes to the regulation of cell proliferation and differentiation that are critical in pathological conditions [15–17]. The effect of IL6ST on the development of IA, however, is still unknown.

This case-control study recruited IA patients and healthy controls to evaluate the diagnostic accuracy and prognostic potential of miR-188-5p for IA. To elucidate the biological function of miR-188-5p in IA pathogenesis, this study established a PDGF-BB-induced VSMC dedifferentiation model. This research sought to assess the clinical significance of miR-188-5p in the diagnosis and prognosis of IA, as well as to elucidate its possible mechanistic contributions to disease development. These findings may offer novel insights for developing effective diagnostic and optimized therapeutic strategies for IA.

## Materials and methods

### Clinical samples and cells

This study cohort comprised 73 IA patients and 79 age- and sex-matched healthy controls at The Fifth People`s Hospital of Jinan between 2021 and 2024. This study was conducted in strict accordance with the ethical principles outlined in the Declaration of Helsinki. The healthy control group comprised individuals who underwent routine physical examinations at The Fifth People’s Hospital of Jinan during the same period as the IA patient recruitment. All enrolled patients completed a standardized follow-up protocol spanning 3 to 60 months to assess clinical recovery and monitor for aneurysm rupture events. The study applied these selection criteria for IA patients:

1. Patients met established diagnostic criteria for unruptured IA confirmed by both CT angiography (CTA) and magnetic resonance imaging (MRI) examinations, were included.
2. Patients who signed an informed consent form were included.
3. Patients with other cardiovascular and cerebrovascular conditions were excluded. This study confirms the absence of clear atherosclerotic plaques or vascular stenosis in the enrolled subjects through detailed medical history reviews and preoperative CTA/MRI imaging assessments.
4. Patients with malignancy, severe immunological disorders, or systemic infections were excluded. By reviewing patients’ medical histories and evaluating laboratory markers of inflammation (such as C-reactive protein and erythrocyte sedimentation rate) as well as autoantibody profiles, this study confirms the absence of significant abnormalities.
5. Patients with impaired heart, liver, or kidney function were excluded.

Expression profiling of miR-188-5p in IA was performed using serum samples. Fasting venous blood samples were drawn in the morning and coagulated at room temperature for 1 h before centrifugation. Serum was isolated by cold centrifugation (3000 × g, 15 min) and maintained at 4 °C.

Human vascular smooth muscle cells (HVSMCs; Pricella, China) were used for the construction of phenotypically transformed VSMCs. The cells were first cultured in complete CM-H116 smooth muscle growth medium (CM-H116 medium + 10% FBS + 100 µg/ml streptomycin + 100 units/ml penicillin; Pricella, China). 20 ng/mL of platelet-derived growth factor BB (PDGF-BB) was used for inducing the phenotypical switching of VSMCs.

After adhering at 37 °C with 5% CO₂ in complete CM-H116 medium overnight, PDGF-BB-treated VSMCs were transfected with the miR-188-5p inhibitor, inhibitor NC, si-IL6ST, or si-NC using Lipofectamine 3000 (Invitrogen, USA).

### The detection of the relationship between miR-188-5p and si-IL6ST

TargetScan revealed the putative miR-188-5p binding site in the IL6ST transcript, which was further functionally confirmed by the dual-luciferase reporter assay. The pmirGLO dual-luciferase reporter vectors containing either wild-type (wt-IL6ST) or mutant (mut-IL6ST) IL6ST 3’UTR sequences were constructed. Renilla luciferase activity served as an internal normalization control for Firefly reporter measurements. At 70–80% confluence in complete CM-H116 medium, HVSMCs were co-transfected using Lipofectamine 3000 with either wt-IL6ST or mut-IL6ST reporter constructs paired with miR-188-5p mimics, miR-188-5p inhibitors, mimic NC or inhibitor NC. Luciferase activity was quantified by measuring firefly and Renilla luminescence using a Multiskan FC (Thermo Fisher Scientific, USA).

### Extraction of total RNA

For the total RNA extraction in serum and cells, TRIzol™ reagent was obtained from Invitrogen, USA. Chloroform performed the phase separation, with isopropanol precipitated the RNA. The RNA pellet was resuspended in RNase-free water after being washed with 75% ethanol and air-dried. A NanoDrop 2000 spectrophotometer (Thermo Fisher Scientific) was used for RNA quantification. Only samples with A₂₆₀/A₂₈₀ ratios ≥ 2.0 were used for downstream applications. All RNA samples were treated with DNase I (RNase-free) for purification. Processed RNA samples were stored at −80 °C until subsequent analysis.

### Quantitative real-time PCR

Hieff Unicon^®^ V Universal Multiplex One Step RT-qPCR Probe Kit (YEASEN, China) and primers for miR-188-5p, IL6ST, alpha smooth muscle actin (α-SMA), smooth muscle protein 22-α (SM22α), matrix metalloproteinase-2 (MMP-2), matrix metalloproteinase-9 (MMP-9), IL-1β, IL-18, tumor necrosis factor-alpha (TNF-α), RNA U6 small nuclear 1 (RNU6-1), and glyceraldehyde-3-phosphate dehydrogenase (GAPDH) were chosen for the reverse transcription quantitative real-time PCR (qPCR). The qPCR was processed in an MX3000P Real-time PCR instrument (Stratagene, Germany). Reaction condition: pre-denaturation at 94 °C for 5 min; denaturation at 94 °C for 20 s; annealing and extension at 62 °C for 40 s; with a total of 40 cycles. Mean cycle threshold (Ct) values were calculated from triplicate technical replicates for each experimental group. Relative gene expression was determined using the 2^−ΔΔCt^ method with RNU6-1 (for miRNA) or GAPDH (for mRNA) normalization.

### Antioxidant activity assay

Malondialdehyde (MDA) levels were quantified using a Lipid Peroxidation MDA Assay Kit (Beyotime, China). Total superoxide dismutase (SOD) activity was measured using a Total Superoxide Dismutase Assay Kit with WST-8 (Beyotime, China), with a spectrophotometric detection at 450 nm.

### Data analysis

Data distribution was assessed using Shapiro-Wilk normality tests (SPSS). Intergroup comparisons employed: Parametric tests (Student’s *t*-test/ANOVA with Bonferroni correction) for normally distributed data; non-parametric alternatives (Wilcoxon rank-sum/Kruskal-Wallis with Dunnett’s post-hoc) for non-normal distributions. Logistic regression analyzed the risk factors for IA. A receiver operating characteristic (ROC) curve was chosen to evaluate the diagnostic utility of miR-188-5p in separating IA patients from healthy controls. Multivariate Cox proportional hazards modeling evaluated clinical predictors of IA rupture. IA rupture probabilities under the different miR-188-5p expression levels were estimated via Kaplan-Meier analysis. Pearson correlation (*r*) quantified miR-188-5p/IL6ST expression relationships.

## Results

### Risk factors for IA

To substantiate the appropriateness of the selected sample size, a priori power analysis was conducted using G*Power software. Assuming a medium effect size (d = 0.5), a significance level of α = 0.05, and a desired statistical power of 1 − β = 0.80, the analysis indicated that a minimum of 64 participants per group was required. Ultimately, the study enrolled 73 IA patients and 79 healthy controls, with both groups exceeding the calculated minimum sample size, thereby fulfilling the fundamental requirements for exploratory research. A subsequent post hoc power analysis confirmed that, under the same parameter settings, the achieved statistical power (1 − β) reached 0.86 for both groups, surpassing the predetermined threshold of 0.80 and aligning with the standards for sample size justification in exploratory clinical investigations.

Comparative analysis revealed no statistically significant differences in baseline characteristics between healthy controls and IA patients (Table 1), with age: 54.76 ± 4.40 years in healthy controls vs. 55.22 ± 3.68 years in IA patients (*P* = 0.488); sex distribution: 40 male and 39 female in healthy controls vs. 32 male and 41 female in IA patients (*P* = 0.402); body mass index (BMI): 22.80 ± 1.72 kg/m² in healthy controls vs. 22.61 ± 1.90 kg/m² in IA patients (*P* = 0.531); drinking status: 33 yes and 46 no in healthy controls vs. 36 yes and 37 no in IA patients (*P* = 0.351); smoking status: 37 yes and 42 no in healthy controls vs. 34 yes and 39 no in IA patients (*P* = 0.552).

**Table 1:** Baseline and clinical characteristics of study subjects

	Controls (*n* = 79)	IA (*n* = 73)	*P*-value
Age (year)	54.76 ± 4.40	55.22 ± 3.68	0.488
Gender (n)			0.402
Male	40	32	
Female	39	41	
BMI (kg/m²)	22.80 ± 1.72	22.61 ± 1.90	0.531
Drinking (n)			0.351
Yes	33	36	
No	46	37	
Smoking (n)			0.552
Yes	37	34	
No	42	39	

*IA* intracranial aneurysm, *BMI* body mass index

**Fig. 1:**
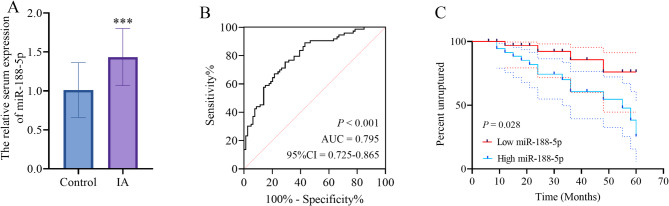
The expression and significance of miR-188-5p in IA. **A** the expression characteristic of miR-188-5p in IA patients. **B** the ROC curve evaluated the performance of miR-188-5p in distinguishing IA patients from healthy controls. **C** the Kaplan-Meier survival curve assessed the significance of miR-188-5p expression in the probability of IA rupture. IA: intracranial aneurysm. ***: *P* < 0.001 vs. the control group

Logistic regression analysis identified miR-188-5p as an risk factor for IA (*P* < 0.001, OR = 7.636, 95% CI = 3.586–16.259, Table 2). Quantitative analysis revealed significantly elevated miR-188-5p expression levels in IA patients compared to healthy controls (Fig. 1A), which further showed a strong diagnostic potential for the disease (*P* < 0.001, AUC = 0.795, 95% CI = 0.725–0.865, Fig. 1B). In this study, the optimal cut-off value was determined to be a relative expression level of 1.28 using the Youden index (J = 0.52). At this threshold, the diagnostic sensitivity of miR-188-5p was 76.71% and the specificity was 67.09%.

**Table 2:** Logistic regression analyzed risk factors for IA

	*P*-value	OR	95% CI
miR-188-5p	< 0.001	7.636	3.586–16.259
Age	0.128	1.772	0.848–3.700
Sex	0.413	1.357	0.654–2.815
BMI	0.625	1.202	0.575–2.512
Drinking	0.126	1.793	0.849–3.786
Smoking	0.627	1.200	0.576–2.498

*OR* odds ratio, *CI* confidence interval, *BMI* body mass index

### Biomarkers for the IA rupture

MiR-188-5p levels correlated strongly with IA rupture risk (HR: 4.454, 95% CI = 1.383–14.343, *P* = 0.012, Table 3), while high miR-188-5p expression (relative expression > 1.43) predicted greater rupture probability (*P* = 0.028, Fig. 1C). Moreover, the aneurysm size (< 5 mm: 39 patients, > 5 mm: 34 patients) was also closely correlated with IA rupture risk (HR: 3.988, 95% CI = 1.280–12.423.280.423, *P* = 0.017, Table 3).

**Table 3:** Multivariate Cox proportional hazards modeling evaluated association between clinical features and IA rupture

	HR factor	95% CI	*P*-value
MiR-188-5p	4.454	1.383–14.343	0.012
Age	1.698	0.622–4.639	0.302
Sex	1.730	0.622–5.380	0.272
BMI	1.275	0.470–3.460	0.633
Drinking	1.250	0.486–3.215	0.643
Smoking	1.337	0.472–3.787	0.584
Aneurysm size	3.988	1.280–12.423	0.017

*IA* intracranial aneurysm, *HR* hazard ratio, *CI* confidence interval, *BMI* body mass index

### The effect of miR-188-5p on VSMCs

**Fig. 2:**
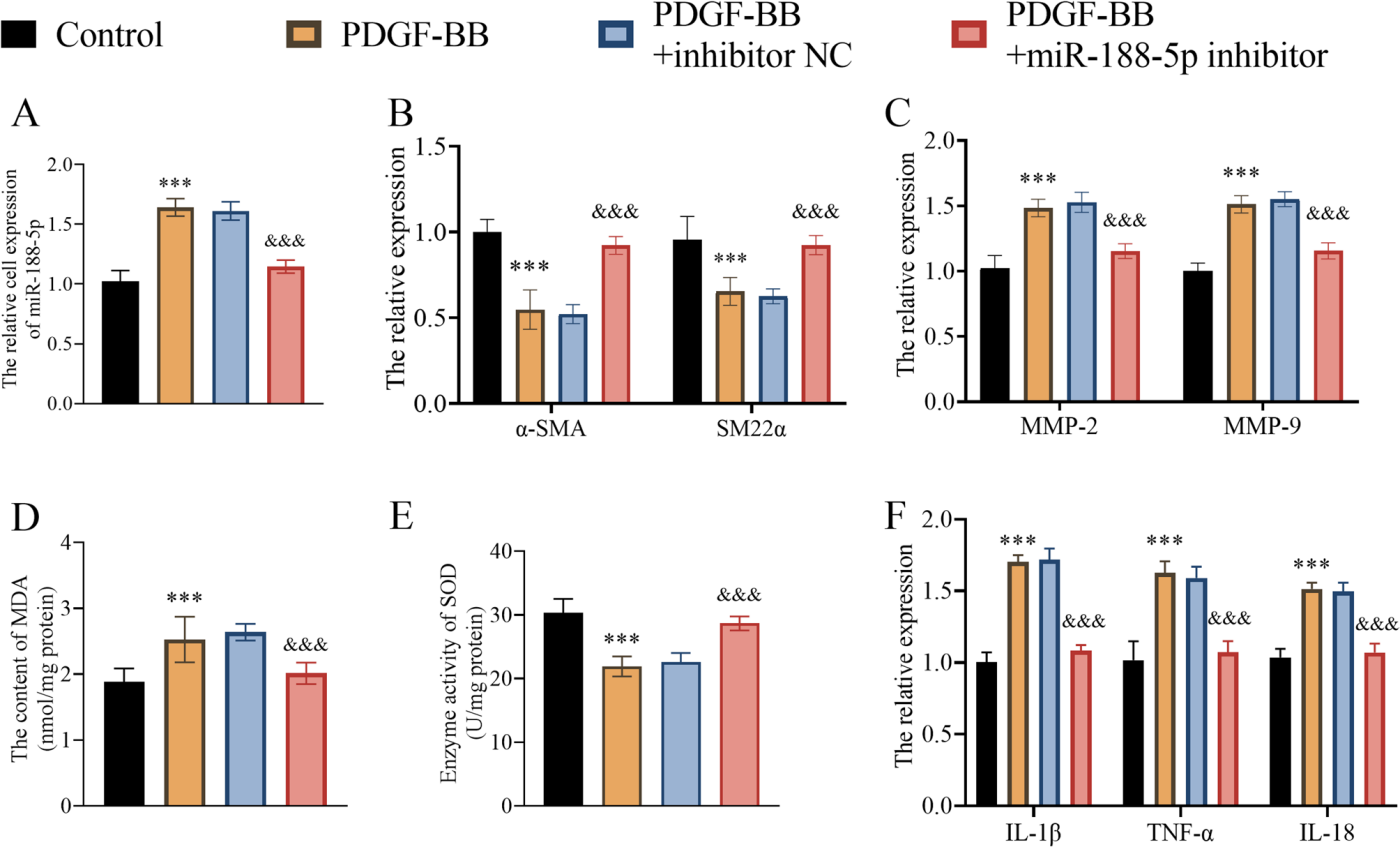
The effect of miR-188-5p on VSMCs. **A** the expression characteristic of miR-188-5p in PDGF-BB-treated VSMCs. **B** the effect of miR-188-5p on the expression of α-SMA and SM22α in VSMCs. **C** the effect of miR-188-5p on the expression of MMP-2 and MMP-9 in VSMCs. D and E, the effect of miR-188-5p on the oxidative stress status including the MDA content (**D**) and SOD enzyme activity (**E**) in VSMC. **F** the effect of miR-188-5p on the proinflammatory cytokine expression, including IL-1β, TNF-α, and IL-18, in VSMC. ***: *P* < 0.001 vs. the control group, &&&: *P* < 0.001 vs. the PDGF-BB-treated group

PDGF-BB treatment showed an elevating effect on the miR-188-5p level in VSMC, which was further diminished by the miR-188-5p inhibitor (Fig. 2A). Compared to the control group, the PDGF-BB treatment caused a lower α-SMA and SM22α expression (Fig. 2B); higher MMP-2 and MMP-9 expression (Fig. 2C); higher oxidative stress, including higher content of MDA (Fig. 2D) and lower enzyme activity of SOD (Fig. 2E); higher proinflammatory cytokine, including IL-1β, TNF-α, IL-18, expression (Fig. 2F) in the VSMCs. The down-regulation of miR-188-5p, caused by miR-188-5p inhibitor, further restored α-SMA and SM22α expression (Fig. 2B), downregulated MMP-2 and MMP-9 expression (Fig. 2C), alleviated oxidative stress (Fig. 2D, E), and downregulated proinflammatory cytokine (Fig. 2F) expression in PDGF-BB-treated VSMCs.

### MiR-188-5p downregulated IL6ST in VSMCs

**Fig. 3:**
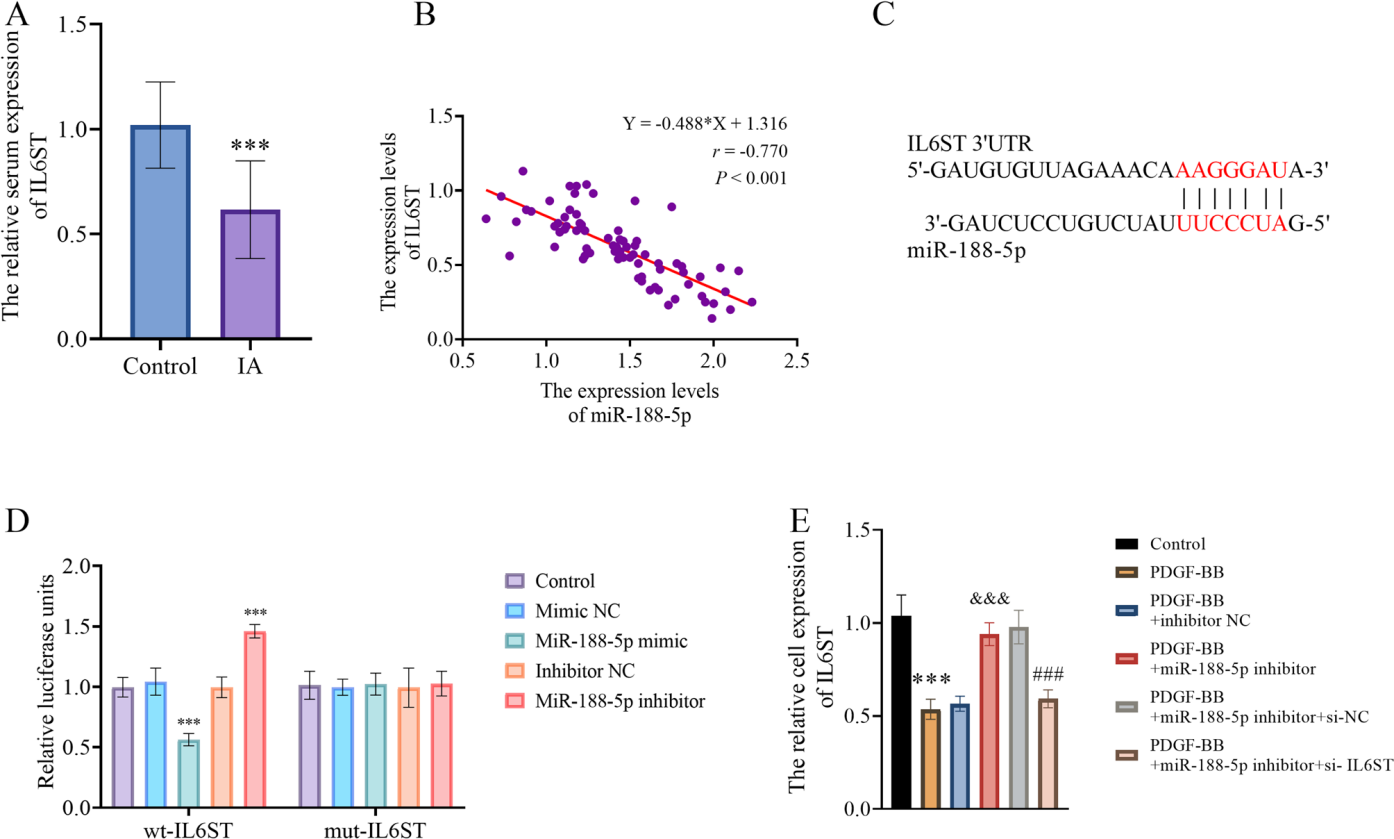
The association between miR-188-5p and IL6ST. **A** the expression characteristic of IL6ST in IA patients. **B** Pearson correlation (*r*) revealed the correlation between miR-188-5p and IL6ST expression in IA. **C** TargetScan identified the potential miR-188-5p binding site in the IL6ST transcript. **D** Dual luciferase assay detected the luciferase activity in wild-type (wt) and mutant (mut) IL6ST groups with up- or downregulation of miR-188-5p. **E** the rescue experiment demonstrated the regulatory effect of miR-188-5p on the IL6ST expression in PDGF-BB-treated VSMC. ***: *P* < 0.001 vs. the control group, &&&: *P* < 0.001 vs. the PDGF-BB-treated group, ###: *P* < 0.001 vs. the PDGF-BB-treated + miR-188-5p inhibitor group

This study revealed a marked reduction in IL6ST expression levels in IA tissue samples versus controls (Fig. 3A, *P* < 0.001), showing a strong inverse correlation with miR-188-5p levels (Fig. 3B, *r* = −0.770, *P* < 0.001). The binding site between miR-188-5p and IL6ST was subsequently predicted using TargetScan (Fig. 3C). The further validation of the binding site between the two biomolecules was through a dual-luciferase reporter assay, the result of which demonstrated that the miR-188-5p up- and downregulation diminished and amplified the luciferase activity in the wt-IL6ST group, respectively (Fig. 3D). In contrast, there was no statistically significant change in the luciferase activity in the mut-IL6ST group with the up- or down-regulation of miR-188-5p (Fig. 3D).

The rescue experiment illustrated that the down-regulation of miR-188-5p rescued down-regulated IL6ST expression in PDGF-BB treated VSMCs (Fig. 3E). The transfection of si-IL6ST further weakened the promotive effect of miR-188-5p-downregulation on IL6ST expression (Fig. 3E).

### IL6ST mediated the effect of miR-188-5p on VSMCs

**Fig. 4:**
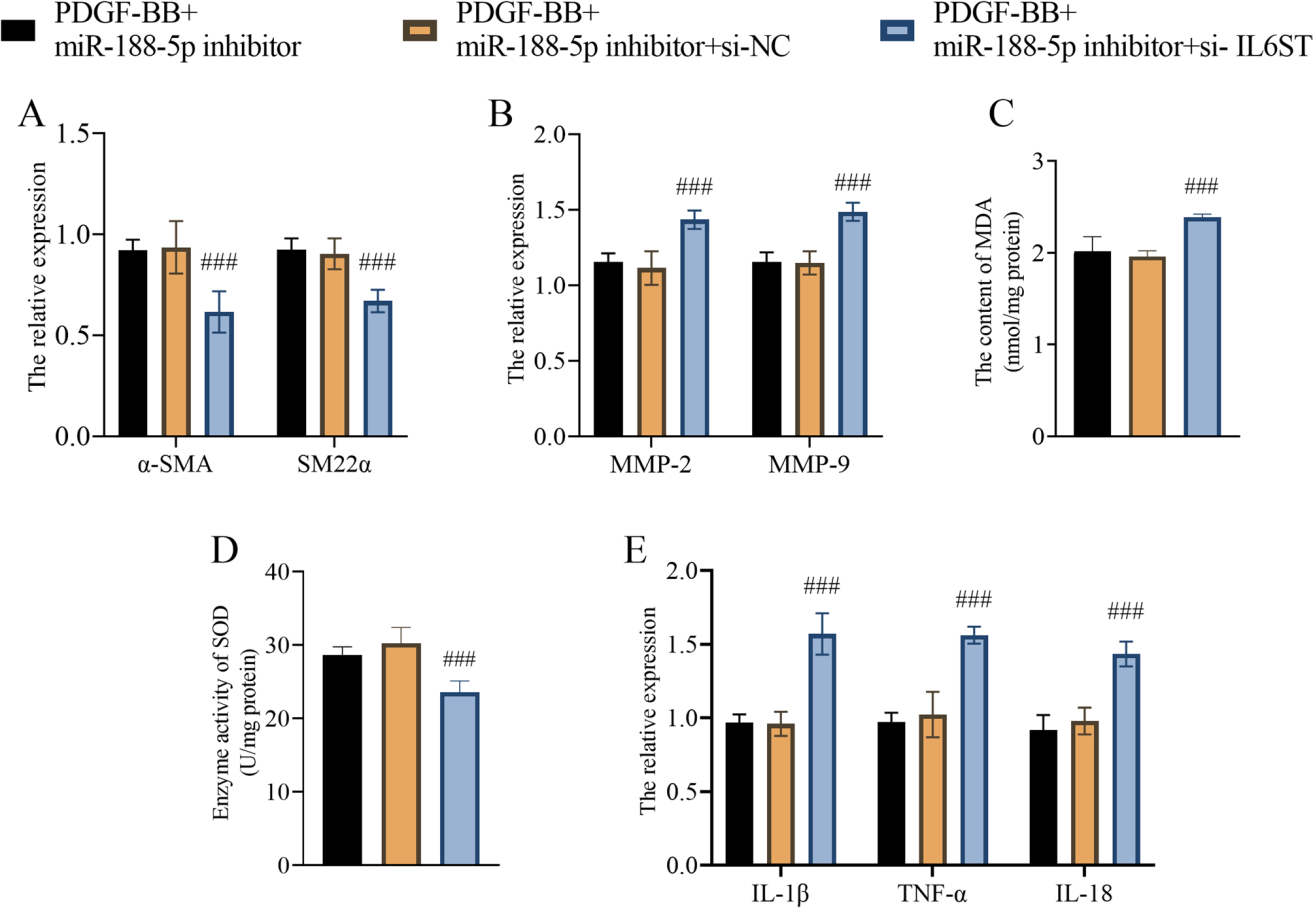
The mechanism of miR-188-5p influencing VSMCs. A-E, IL6ST mediated the effect of miR-188-5p on VSMC α-SMA and SM22α expression (**A**), MMP-2 and MMP-9 expression (**B**), oxidative stress status including the MDA content (**C**) and SOD enzyme activity (**D**), and proinflammatory cytokine expression, including IL-1β, TNF-α, and IL-18 (**E**). ###: *P* < 0.001 vs. the PDGF-BB-treated + miR-188-5p inhibitor group

The rescue experiments showed that the downregulation of IL6ST induced by si-IL6ST further diminished the effect of miR-188-5p downregulation on phenotypical switched VSMCs, including inhibited α-SMA and SM22α expression (Fig. 4A), restored MMP-2 and MMP-9 expression (Fig. 4B). recovered oxidative stress (Fig. 4C, D), and facilitated proinflammatory cytokine (Fig. 4E).

## Discussion

IAs primarily develop due to the degeneration of the arterial elastic layer and muscular layer [18]. The rupture of such aneurysms poses a significant life-threatening risk to patients [19, 20]. For individuals diagnosed with IA, prompt detection is paramount, while the development of an appropriate and effective treatment approach is crucial for ensuring favorable patient outcomes. Emerging evidence has demonstrated that miRNAs serve as critical regulators of gene expression and protein function, exhibiting substantial diagnostic, prognostic and therapeutic potential across diverse diseases [21, 22]. In this study, miR-188-5p showed great potential in IA diagnosis, which was consistent with a previous study that reported an abnormal miR-188-5p upregulation in IA [11]. It was suggested that miR-188-5p serves as a key regulatory molecule in the pathogenesis of diverse cardiovascular diseases. MiR-188-5p promotes cellular senescence in the aneurysm wall, potentially regulating macrophage and T cell senescence to contribute to the pathogenesis of AAA [23]. In addition to the cardiovascular disease, overexpression of miR-188 in endothelial cells (ECs) has been demonstrated to contribute to the age-related decline of type H vessels, a specialized vascular subtype critical for bone homeostasis [24]. Vascular inflammation triggered by EC injury represents a pivotal pathogenic mechanism contributing to both the initiation and progression of aortic aneurysm and dissection (AAD) [25]. Based on our findings, we speculated that miR-188-5p may also play a significant role in IA pathogenesis, giving it diagnostic value for this condition. As an exploratory study, this research aimed to assess miR-188-5p’s standalone diagnostic value and did not directly compare it to established IA biomarkers (e.g., circulating MMP-9, IL-6); future studies will include these biomarkers for comparing AUC/combined diagnostic performance and clarifying miR-188-5p’s clinical incremental value. Additionally, current evaluation of miR-188-5p provides a core biomarker basis for a future multi-factor model—integrating its expression with clinical parameters (e.g., aneurysm size, patient age) to build a “molecular + clinical” combined model and boost clinical practicality via improved diagnostic accuracy. This study excluded abdominal aortic aneurysms, atherosclerosis and bone metabolism disorders to reduce confounding, and found miR-188-5p’s IA mechanism (downregulating IL6ST to modulate VSMC transformation) differs from that in abdominal aortic aneurysms (cellular senescence) and bone metabolism (type H vessels)—providing preliminary IA-specific evidence; future studies will add controls (abdominal aortic aneurysms, coronary heart disease, osteoporosis) to test serum miR-188-5p, develop a “miR-188-5p + IL6ST/α-SMA” model, assess its IA-discriminating ability via ROC, and validate with multicenter samples to clarify its scope as an IA biomarker.

Except for its diagnostic value, miR-188-5p also showed a remarkable prognostic value for IA. Along with the aneurysm size, a higher miR-188-5p was a predictor for IA rupture. The increasing detection of unruptured IAs has intensified debate regarding optimal management strategies, primarily due to persistent uncertainties in rupture risk [26, 27]. This clinical dilemma carries significant implications, as IA rupture represents the leading etiology of SAH, an event associated with high mortality and severe disability among survivors [28]. This study provides a novel perspective in stratifying rupture risk in unruptured IAs, highlighting the potential of modulating miR-188-5p expression as a preventive strategy against IA rupture. Specifically, for patients with high miR-188-5p expression, even if the aneurysm (traditionally low-risk) is < 5 mm, CTA/MRI follow-up can be shortened from 12 to 3–6 months for closer monitoring; for those with high expression and aneurysm > 5 mm, it may aid prioritizing interventional therapy (e.g., endovascular coiling). It complements the current “aneurysm size + comorbidities + symptoms” framework, helping clinicians balance conservative vs. interventional risks and improve personalized treatment precision. The rupture of IA is primarily driven by progressive VSMC dysfunction and extracellular matrix (ECM) dysregulated degradation [29, 30]. To explore the mechanistic role of miR-188-5p in modulating IA rupture risk, this study investigated its regulatory effects on VSMCs.

This study employed a PDGF-BB-induced VSMC dedifferentiation model [31], given the established role of VSMC phenotypic switching in aneurysm pathogenesis [32]. This study found that miR-188-5p mediates PDGF-BB-driven VSMC dedifferentiation, marked by reduced α-SMA and SM22α expression. The two biomolecules are key VSMC markers, the downregulation of which serves as a biomarker for human aneurysms [33]. Furthermore, miR-188-5p enhanced MMP-2 and MMP-9 expression in dedifferentiated VSMCs. The two biomolecules are key matrix-degrading enzymes implicated in aneurysm pathogenesis through extracellular matrix (ECM) disruption [34]. Genetic knockdown of MMP-2 and MMP-9 significantly attenuated IA progression in the mouse IA model [35]. Additionally, miR-188-5p was further shown to induce oxidative stress and stimulate proinflammatory cytokine expression in VSMCs. Substantial evidence demonstrates that oxidative stress induces inflammatory activation and subsequent cell injury [36]. In IA, excessive free radicals can activate MMPs, subsequently inducing vascular wall remodeling and eventual structural breakdown [37]. Proinflammatory cytokines compromise arterial homeostasis by driving inflammatory responses and triggering apoptotic pathways in vascular cells, which progressively weaken vessel wall integrity and facilitate aneurysm progression [38–40]. The results demonstrated a promotive effect of miR-188-5p on VSMC phenotypic switching and functional impairment. These findings suggested that the way miR-188-5p contributed to IA formation and rupture might be driving VSMC dedifferentiation and dysfunction. This study provided novel mechanistic insights into the role of miR-188-5p as both a potential biomarker and active contributor in IA pathogenesis.

While miRNAs are known to regulate disease pathogenesis through gene expression modulation, our study specifically reveals that miR-188-5p impaired VSMC function via IL6ST downregulation. Functioning as a critical cytokine signal transducer, IL6ST plays a pivotal role in mediating IL-6- and OSM-induced cellular responses [41, 42]. This study suggested that miR-188-5p-mediated IL6ST downregulation may disrupt IL-6- and OSM-induced cellular responses in VSMCs, potentially contributing to IA progression. In addition, IL6ST mediated the activation of signal pathways that involved signal transducer and activator of transcription (STAT) [43], mitogen-activated protein kinase (MAPK) [44], and phosphatidylinositol 3-kinase (PI3K)/protein kinase B (Akt) [44], from which IL6ST gains the power of regulating cell proliferation, migration, apoptosis and inflammation [45]. This study identified that miR-188-5p might be a regulator of VSMC activity through IL6ST modulation, revealing its potential pathogenic role in IA progression. The miR-188-5p/IL6ST axis emerges as a potential therapeutic target for IA management.

Although our findings highlight the clinical relevance of miR-188-5p for IA diagnosis and management, this study has certain limitations. First, the patient population, sourced from a single research institution, may exhibit concentrated demographic or clinical characteristics—such as regional dietary habits and a relatively uniform distribution of underlying diseases—which could limit the generalizability of the findings across different regions and populations. To mitigate this limitation, the research team has established collaboration agreements with multiple clinical centers and plans to conduct multicenter prospective cohort studies in the future. These efforts aim to enhance the external validity of the results by expanding the diversity of the sample population. Additionally, stratified analyses will be performed based on key variables such as age, aneurysm size, and comorbidities to evaluate the applicability of miR-188-5p across distinct patient subgroups. Second, we have only identified the role of miR-188-5p in IA pathogenesis through in vitro experiments, which limited our understanding of its precise mechanistic pathways in vivo. Due to constraints in experimental conditions, the model development during the mechanistic exploration phase of this study did not achieve the intended objectives. The research team has adopted targeted improvements: collaborating with an animal experimentation expert group, using an optimized elastase-induced + spontaneously hypertensive rat (SHR) modeling method (adjusting elastase dosage to boost aneurysm formation rate to > 60% for statistical/methodological standards), and integrating qPCR/immunohistochemistry to detect miR-188-5p, IL6ST, VSMC phenotypic transitions, extracellular matrix degradation, oxidative stress, and inflammation-related molecules—all to provide solid in vivo evidence for miR-188-5p’s role in IA initiation/progression. Furthermore, this study is limited by a single-center sample and few recorded IA rupture events during follow-up, which may reduce statistical estimation precision. To address this, future research will follow the proposed multicenter design: enroll more diverse samples, increase recorded rupture outcomes, better control potential confounders (e.g., subclinical atherosclerosis) not fully addressed earlier, and re-run Cox proportional hazards regression. These steps aim to improve statistical power, narrow confidence intervals, validate miR-188-5p’s robustness for IA rupture prediction, and boost result reliability to support its clinical applicability.

## Conclusion

This study identified elevated miR-188-5p expression as both a diagnostic biomarker for IA and an indicator of rupture risk. Mechanistically, miR-188-5p promoted VSMC phenotypic switching and functional impairment through targeted downregulation of IL6ST, thereby accelerating IA progression. These findings position the miR-188-5p/IL6ST axis as a promising therapeutic target for IA management and rupture prevention.

## Data Availability

Corresponding authors may provide data and materials.
